# A 2:1 cocrystal of the *cis* and *trans* isomers of bis­[1,1,1,5,5,5-hexa­fluoro­pentane-2,4-dionato(1−)-κ^2^ *O*,*O*′]bis­(4-phenyl­pyridine *N*-oxide-κ*O*)copper(II)

**DOI:** 10.1107/S1600536810049196

**Published:** 2010-11-27

**Authors:** José A. Fernandes, Ana I. Ramos, Susana S. Braga, Filipe A. Almeida Paz

**Affiliations:** a Department of Chemistry, University of Aveiro, CICECO, 3810-193 Aveiro, Portugal

## Abstract

The title compound is a co-crystal of the *cis* and *trans* isomers, namely *cis*-bis­[1,1,1,5,5,5-hexa­fluoro­pentane-2,4-dionato(1−)-κ^2^ *O*,*O′*]bis­(4-phenyl­pyridine *N*-oxide-κ*O*)copper(II)–*trans*-bis­[1,1,1,5,5,5-hexa­fluoro­pentane-2,4-dionato(1−)-κ^2^ *O*,*O′*]bis(4-phenyl­pyridine *N*-oxide-κ*O*)copper(II) (2/1), [Cu(C_5_HF_6_O_2_)_2_(C_11_H_9_NO)_2_]. In both isomers, the coordination geometry of the Cu^2+^ atom is octa­hedral, exhibiting typical Jahn–Teller distortion. The metal atom of the *trans* isomer is located on an inversion centre. In the *cis* isomer, the phenyl ring in one 4-phenyl­pyridine *N*-oxide ligand is disordered over two orientations in a 1:1 ratio. In the crystal, weak inter­molecular C—H⋯F and C—H⋯O contacts establish connections between the *cis* and *trans* isomers.

## Related literature

For crystal structures with 4-phenyl­pyridine-*N*-oxide, see: Papadaki *et al.* (1999 ▶); Watson & Johnson (1971 ▶); Verdejo *et al.* (2009 ▶); Ramos *et al.* (2010 ▶). For general background studies on cyclo­dextrin inclusion compounds from our research group, see: Marques *et al.* (2008 ▶, 2009 ▶); Petrovski *et al.* (2008 ▶); Pereira *et al.* (2006 ▶, 2008 ▶); Braga *et al.* (2006 ▶). For a description of the Cambridge Structural Database, see: Allen (2002 ▶). For a description of the graph-set notation for hydrogen-bonded aggregates, see: Grell *et al.* (1999 ▶).
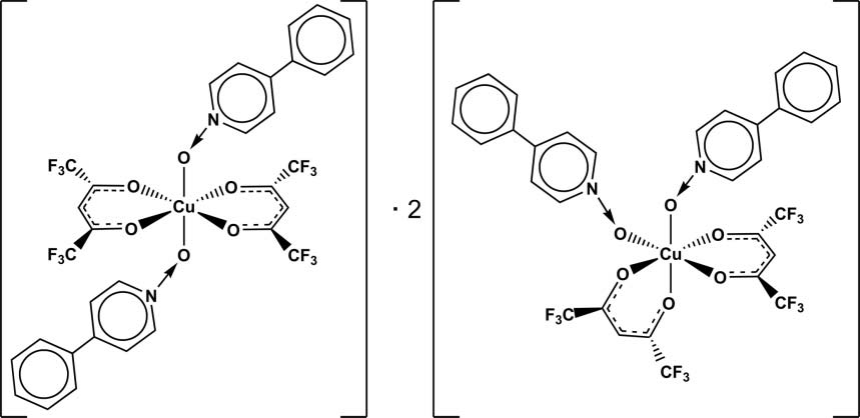


## Experimental

### 

#### Crystal data

[Cu(C_5_HF_6_O_2_)_2_(C_11_H_9_NO)_2_]
*M* *_r_* = 820.04
Triclinic, 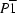

*a* = 14.3902 (6) Å
*b* = 14.7372 (6) Å
*c* = 14.9636 (10) Å
α = 102.191 (3)°
β = 111.192 (3)°
γ = 114.122 (2)°
*V* = 2448.4 (2) Å^3^
*Z* = 3
Mo *K*α radiation
μ = 0.79 mm^−1^
*T* = 150 K
0.20 × 0.16 × 0.10 mm

#### Data collection

Bruker X8 Kappa CCD APEXII diffractometer
Absorption correction: multi-scan (*SADABS*; Sheldrick, 1998 ▶) *T*_min_ = 0.859, *T*_max_ = 0.926
128806 measured reflections
13039 independent reflections
8907 reflections with *I* > 2σ(*I*)
*R*_int_ = 0.052

#### Refinement

*R*[*F* ^2^ > 2σ(*F* ^2^)] = 0.054
*wR*(*F* ^2^) = 0.186
*S* = 0.97
13039 reflections
702 parameters
H-atom parameters constrained
Δρ_max_ = 1.18 e Å^−3^
Δρ_min_ = −0.76 e Å^−3^

### 

Data collection: *APEX2* (Bruker, 2006 ▶); cell refinement: *SAINT-Plus* (Bruker, 2005 ▶); data reduction: *SAINT-Plus*; program(s) used to solve structure: *SHELXTL* (Sheldrick, 2008 ▶); program(s) used to refine structure: *SHELXTL*; molecular graphics: *DIAMOND* (Brandenburg, 2009 ▶); software used to prepare material for publication: *SHELXTL*.

## Supplementary Material

Crystal structure: contains datablocks global, I. DOI: 10.1107/S1600536810049196/cv5006sup1.cif

Structure factors: contains datablocks I. DOI: 10.1107/S1600536810049196/cv5006Isup2.hkl

Additional supplementary materials:  crystallographic information; 3D view; checkCIF report

## Figures and Tables

**Table 1:** Hydrogen-bond geometry (Å, °)

*D*—H⋯*A*	*D*—H	H⋯*A*	*D*⋯*A*	*D*—H⋯*A*
C41—H41⋯F2^i^	0.95	2.45	3.219 (5)	138
C45′—H45*B*⋯F4^ii^	0.95	2.31	3.159 (2)	148
C42—H42⋯O3^i^	0.95	2.47	3.376 (3)	160
C6—H6⋯O9^iii^	0.95	2.27	3.215 (3)	171
C31—H31⋯O8^iv^	0.95	2.41	3.333 (5)	163
C27—H27⋯O6	0.95	2.55	3.389 (4)	147
C38—H38⋯O4	0.95	2.55	3.334 (6)	140
C10—H10⋯O2^ii^	0.95	2.51	3.249 (6)	134

Symmetry codes: (i) 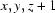; (ii) 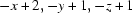; (iii) 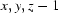; (iv) 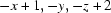.

**Fig. 1.**
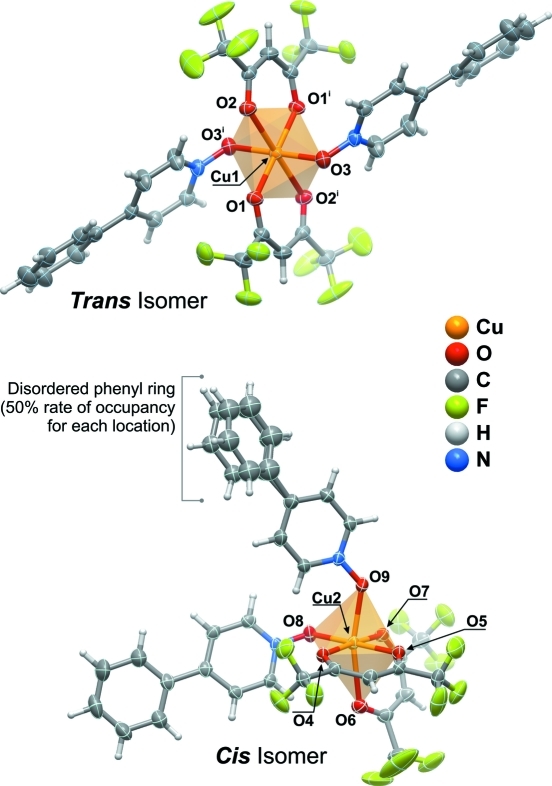
Schematic representation of the two distinct cis and trans isomers present in the title compound. All non-hydrogen atoms are represented as thermal ellipsoids drawn at the 50% probability level and hydrogen atoms as small spheres with arbitrary radius. The labeling scheme is provided for all atoms composing the first coordination sphere of Cu1 and Cu2. Symmetry transformation used to generate equivalent atoms: (i) 2 - x, 1 - y, 1 - z.

**Fig. 2.**
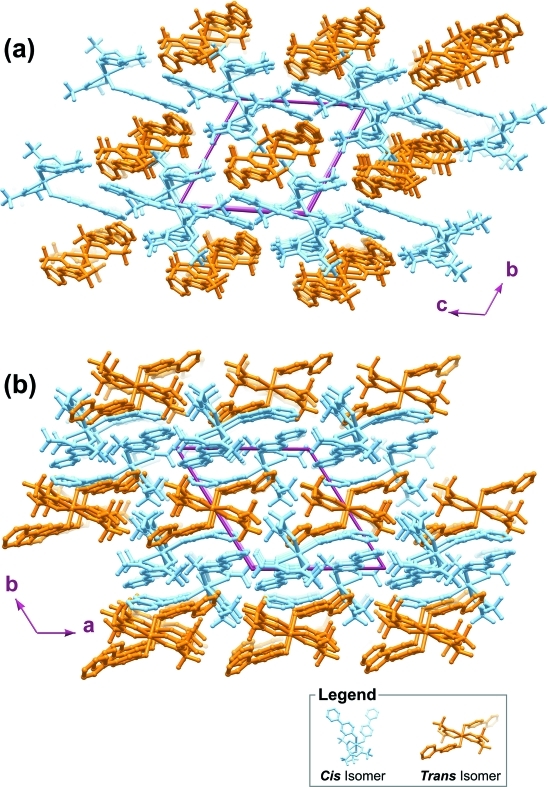
Crystal packing of the title compound viewed in perspective along the (a) [100] and (b) [001] directions of the unit cell. H-atoms have been omitted for clarity and the two distinct cis and trans isomers are represented in different colour.

## supplementary crystallographic information

### Comment

The coordination chemistry of 4-phenylpyridine-*N*-oxide (PPNO; C_11_H_9_NO) is rather unknown. Surveying the Cambridge Structural Database (Allen, 2002) only four structures were found: copper (Watson & Johnson, 1971) and tin complexes (Papadaki *et al.*, 1999), an inclusion compound of PPNO into a derivative of calix[4]pyrrole (Verdejo *et al.*,2009), and a recently published Cu^2+^ dinuclear complex (Ramos *et al.*, 2010). Following our interest in the preparation and study of the properties of inclusion compounds of cyclodextrins (Marques *et al.*, 2009; Petrovski *et al.*, 2008; Pereira *et al.*, 2008; Marques *et al.*, 2008; Pereira *et al.*, 2006; Braga *et al.*, 2006) we prepared a new copper compound suitable for being used as a guest in inclusion chemistry. We have reacted [Cu(hfac)_2_] (where hfac^-^ stands for hexafluoroacetylacetonate) with PPNO, affording the title compound as deep-green crystals, whose structure we wish to report here.

The title compound (see Scheme) results from a co-crystallization of the *cis* and *trans* isomers of [Cu(hfac)_2_(PPNO)_2_] (Figure 1). For each isomer, the Cu^2+^ centre is coordinated to two hfac^-^ and two PPNO ligands, with the {CuO_6_} coordination polyhedra resembling the typical Jahn-Teller distorted octahedral geometry.

In the *trans* isomer (Figure 1 - top) the metal centre is situated at an inversion point. The Cu1—O distances are 1.966 (2) and 2.331 (2) Å for the hfac^-^ anion, and 1.968 (2) Å for the PPNO ligand. The *cis* octahedral angles fall within a short range of the ideal value being found in the 86.25 (9)–93.75 (9)° range. In the *cis* isomer (Figure 1 - bottom) the Cu2—O equatorial distances range from 1.9532 (19) to 1.990 (2) Å, while the apical distances are 2.230 (Cu2—O9 with PPNO) and 2.365 (2) Å (Cu—O6 with hfac^-^). As in the other isomer, the *cis* and *trans* octahedral angles in this complex also approach those of an ideal octahedron being found in the 80.26 (9)–98.43 (9)° and 165.15 (8)–176.63 (9)° ranges, respectively. In addition, the terminal phenyl ring of one coordinated PPNO was found to be disordered over two positions (see Experimental Section). This crystallographic feature seems to be driven by the need to form a short C—H···F contact (see below). An interesting feature common to both isomers concerns the 〈(Cu—O—N) angles of the coordinated PPNO ligands which approach *ca* 120°. Indeed, while the 〈(Cu1—O3—N1) angle for the *trans* isomer is 117.82 (18)°, for the *cis* isomer the analogues 〈(Cu1—O9—N3) and 〈(Cu1—O8—N2) angles are 131.12 (17) and 122.39 (16)°, respectively.

Besides the need to effectively fill the available space, the crystal packing of the two isomers (Figure 2) is also mediated by a number of C—H···F and C—H···O short contacts (Table 1). The shortest of the intermolecular contacts concerns the *para* H-atom of one of the disordered phenyl ring of the *cis* isomer, with a H···F distance of *ca* 2.31 Å [C45'—H45B···F4 angle of *ca* 148°]. While the C31—H31···O8 contact leads to connections between adjacent *cis* isomers, the combination of C6—H6···O9 and C42—H42···O3 interactions connects instead neighbouring *cis* and *trans* isomers [both form *R^2^_2_(8)* graph set motifs - Grell *et al.* (1999)].

### Experimental

All chemicals and solvents were purchased from commercial sources and were used without further purification.

4-Phenylpyridine-*N*-oxide (PPNO, Aldrich, 71.1 mg, 0.415 mmol) was slowly added to a previously prepared 10 ml solution of [Cu(hfac)_2_] (99.6 mg, 0.208 mmol) in acetone (hfac^-^ = hexafluoroacetylacetonate). The resulting solution was allowed to homogenize with magnetic stirring at 30 °C for 60 minutes, after which time the solvent was evaporated. Diffusion of an ethanolic solution of the extract into water afforded two crystalline solids at different crystallization times. The first compound to crystallize, and obtained in smaller quantity as small light-green single crystals, was identified as identical to the binuclear species [Cu(C_5_HF_6_O_2_)_2_(C_11_H_9_NO)]_2_ recently described by us (Ramos *et al.*, 2010). The second material, obtained at a later stage, was solely composed of large deep-green block crystals of the title compound.

### Refinement

Hydrogen atoms bound to aromatic carbon atoms were placed in their idealized positions and were included in the final structural model in riding-motion approximation with C—H = 0.95 Å. The isotropic thermal displacement parameters for these atoms were fixed at 1.2×*U*_eq/iso_ of the respective parent carbon atom.

The phenyl ring of one coordinated PPNO ligand in the *cis* isomer was treated as disordered over two orientations with occupancies fixed to 0.5. The carbon atoms were included in the final structural model and allowed to refine unrestrained. An independent and refineable *U*_iso_ value was modelled for each position of this phenyl ring.

### Crystal data

**Table:**

[Cu(C_5_HF_6_O_2_)_2_(C_11_H_9_NO)_2_]	*Z* = 3
*M**_r_* = 820.04	*F*(000) = 1233
Triclinic, *P*1	*D*_x_ = 1.668 Mg m^−^^3^
Hall symbol: -P 1	Mo *K*α radiation, λ = 0.71073 Å
*a* = 14.3902 (6) Å	Cell parameters from 9410 reflections
*b* = 14.7372 (6) Å	θ = 2.9–25.7°
*c* = 14.9636 (10) Å	µ = 0.79 mm^−^^1^
α = 102.191 (3)°	*T* = 150 K
β = 111.192 (3)°	Block, green
γ = 114.122 (2)°	0.20 × 0.16 × 0.10 mm
*V* = 2448.4 (2) Å^3^	

### Data collection

**Table:**

Bruker X8 Kappa CCD APEXII diffractometer	13039 independent reflections
Radiation source: fine-focus sealed tube	8907 reflections with *I* > 2σ(*I*)
graphite	*R*_int_ = 0.052
ω / φ scans	θ_max_ = 29.1°, θ_min_ = 3.5°
Absorption correction: multi-scan (*SADABS*; Sheldrick, 1998)	*h* = −19→19
*T*_min_ = 0.859, *T*_max_ = 0.926	*k* = −20→20
128806 measured reflections	*l* = −20→20

### Refinement

**Table:**

Refinement on *F*^2^	Primary atom site location: structure-invariant direct methods
Least-squares matrix: full	Secondary atom site location: difference Fourier map
*R*[*F*^2^ > 2σ(*F*^2^)] = 0.054	Hydrogen site location: inferred from neighbouring sites
*wR*(*F*^2^) = 0.186	H-atom parameters constrained
*S* = 0.97	*w* = 1/[σ^2^(*F*_o_^2^) + (0.1201*P*)^2^ + 1.7138*P*] where *P* = (*F*_o_^2^ + 2*F*_c_^2^)/3
13039 reflections	(Δ/σ)_max_ = 0.001
702 parameters	Δρ_max_ = 1.18 e Å^−^^3^
0 restraints	Δρ_min_ = −0.76 e Å^−^^3^

### Special details

**Table:**

Geometry. All e.s.d.'s (except the e.s.d. in the dihedral angle between two l.s. planes) are estimated using the full covariance matrix. The cell e.s.d.'s are taken into account individually in the estimation of e.s.d.'s in distances, angles and torsion angles; correlations between e.s.d.'s in cell parameters are only used when they are defined by crystal symmetry. An approximate (isotropic) treatment of cell e.s.d.'s is used for estimating e.s.d.'s involving l.s. planes.
Refinement. Refinement of *F*^2^ against ALL reflections. The weighted *R*-factor *wR* and goodness of fit *S* are based on *F*^2^, conventional *R*-factors *R* are based on *F*, with *F* set to zero for negative *F*^2^. The threshold expression of *F*^2^ > σ(*F*^2^) is used only for calculating *R*-factors(gt) *etc*. and is not relevant to the choice of reflections for refinement. *R*-factors based on *F*^2^ are statistically about twice as large as those based on *F*, and *R*- factors based on ALL data will be even larger.

### Fractional atomic coordinates and isotropic or equivalent isotropic displacement parameters (Å^2^)

**Table:**

	*x*	*y*	*z*	*U*_iso_*/*U*_eq_	Occ. (<1)
Cu1	1.0000	0.5000	0.5000	0.02630 (13)	
O1	1.06522 (18)	0.43056 (18)	0.43599 (16)	0.0319 (5)	
C1	1.1327 (3)	0.3990 (2)	0.4770 (2)	0.0287 (6)	
C2	1.1518 (3)	0.3376 (3)	0.3965 (3)	0.0361 (7)	
F1	1.1926 (2)	0.3978 (2)	0.3496 (2)	0.0584 (6)	
F2	1.04860 (18)	0.24671 (17)	0.31959 (15)	0.0458 (5)	
F3	1.2244 (2)	0.3043 (2)	0.43635 (17)	0.0538 (6)	
O2	0.88041 (19)	0.49436 (18)	0.34165 (16)	0.0328 (5)	
C3	0.8246 (2)	0.5399 (2)	0.3395 (2)	0.0285 (6)	
C4	0.8118 (3)	0.5900 (3)	0.4212 (2)	0.0337 (7)	
H4	0.7618	0.6179	0.4054	0.040*	
C5	0.7617 (3)	0.5407 (3)	0.2320 (3)	0.0359 (7)	
F4	0.8281 (3)	0.5615 (5)	0.1899 (3)	0.1379 (19)	
F5	0.7333 (3)	0.6139 (2)	0.23628 (19)	0.0926 (12)	
F6	0.6668 (3)	0.4474 (2)	0.1644 (2)	0.0902 (11)	
O3	0.88192 (19)	0.35102 (18)	0.46898 (17)	0.0354 (5)	
N1	0.8109 (2)	0.3408 (2)	0.5094 (2)	0.0320 (5)	
C6	0.6972 (3)	0.3031 (3)	0.4449 (2)	0.0331 (6)	
H6	0.6687	0.2863	0.3718	0.040*	
C7	0.6221 (3)	0.2888 (3)	0.4845 (2)	0.0347 (7)	
H7	0.5419	0.2617	0.4379	0.042*	
C8	0.6610 (3)	0.3131 (2)	0.5910 (2)	0.0321 (6)	
C9	0.7791 (3)	0.3525 (4)	0.6541 (3)	0.0509 (10)	
H9	0.8101	0.3704	0.7275	0.061*	
C10	0.8521 (3)	0.3661 (4)	0.6130 (3)	0.0519 (10)	
H10	0.9329	0.3936	0.6581	0.062*	
C11	0.5800 (3)	0.2971 (3)	0.6340 (3)	0.0331 (6)	
C12	0.4599 (3)	0.2231 (3)	0.5692 (3)	0.0461 (8)	
H12	0.4289	0.1828	0.4961	0.055*	
C13	0.3838 (4)	0.2068 (4)	0.6096 (4)	0.0623 (11)	
H13	0.3015	0.1564	0.5640	0.075*	
C14	0.4282 (4)	0.2639 (4)	0.7156 (4)	0.0588 (11)	
H14	0.3768	0.2518	0.7437	0.071*	
C15	0.5464 (4)	0.3380 (3)	0.7804 (3)	0.0506 (9)	
H15	0.5766	0.3782	0.8533	0.061*	
C16	0.6230 (4)	0.3552 (3)	0.7410 (3)	0.0409 (8)	
H16	0.7050	0.4068	0.7871	0.049*	
Cu2	0.42260 (3)	0.14398 (3)	1.13094 (3)	0.02492 (11)	
O4	0.39585 (18)	0.21929 (16)	1.04058 (15)	0.0273 (4)	
O5	0.45638 (19)	0.26248 (17)	1.25349 (16)	0.0305 (4)	
C17	0.3539 (3)	0.3347 (2)	0.9737 (2)	0.0321 (6)	
C18	0.3872 (2)	0.3017 (2)	1.0654 (2)	0.0268 (6)	
C19	0.4018 (3)	0.3605 (2)	1.1592 (2)	0.0315 (6)	
H19	0.3879	0.4186	1.1647	0.038*	
C20	0.4367 (3)	0.3365 (2)	1.2465 (2)	0.0288 (6)	
C21	0.4549 (3)	0.4082 (3)	1.3494 (3)	0.0390 (7)	
F7	0.2516 (2)	0.25163 (18)	0.88963 (17)	0.0565 (6)	
F8	0.3423 (2)	0.42005 (18)	0.99617 (17)	0.0525 (6)	
F9	0.4342 (2)	0.3597 (2)	0.94430 (19)	0.0566 (6)	
F10	0.5677 (2)	0.4697 (2)	1.4209 (2)	0.0867 (10)	
F11	0.4073 (3)	0.3469 (2)	1.3926 (2)	0.0623 (7)	
F12	0.4072 (3)	0.4666 (2)	1.3366 (2)	0.0767 (9)	
O6	0.2300 (2)	0.07746 (19)	1.10322 (18)	0.0371 (5)	
O7	0.43470 (18)	0.05692 (18)	1.21351 (16)	0.0303 (4)	
C22	0.1066 (4)	0.0501 (4)	1.1753 (4)	0.0550 (10)	
C23	0.2088 (3)	0.0465 (3)	1.1671 (3)	0.0338 (7)	
C24	0.2666 (3)	0.0098 (3)	1.2351 (3)	0.0404 (8)	
H24	0.2335	−0.0191	1.2746	0.048*	
C25	0.3675 (3)	0.0134 (2)	1.2473 (2)	0.0301 (6)	
C26	0.4132 (3)	−0.0390 (3)	1.3165 (3)	0.0406 (8)	
F13	0.1122 (5)	0.1406 (5)	1.1744 (5)	0.153 (2)	
F14	0.0074 (3)	−0.0252 (6)	1.1000 (4)	0.187 (3)	
F15	0.1073 (3)	0.0515 (4)	1.2628 (3)	0.1043 (13)	
F16	0.4315 (3)	−0.1092 (2)	1.2652 (2)	0.0679 (7)	
F17	0.5183 (2)	0.0357 (2)	1.40156 (18)	0.0620 (6)	
F18	0.3441 (3)	−0.0901 (3)	1.3506 (3)	0.0893 (11)	
O8	0.3869 (2)	0.02132 (18)	1.01409 (16)	0.0337 (5)	
N2	0.3308 (2)	0.00417 (19)	0.91308 (19)	0.0280 (5)	
C27	0.2274 (3)	−0.0005 (2)	0.8720 (2)	0.0308 (6)	
H27	0.1966	0.0132	0.9161	0.037*	
C28	0.1671 (3)	−0.0250 (2)	0.7668 (2)	0.0317 (6)	
H28	0.0959	−0.0258	0.7394	0.038*	
C29	0.2082 (3)	−0.0491 (2)	0.6987 (2)	0.0300 (6)	
C30	0.3160 (3)	−0.0418 (3)	0.7462 (2)	0.0321 (6)	
H30	0.3486	−0.0561	0.7039	0.039*	
C31	0.3757 (3)	−0.0147 (2)	0.8518 (2)	0.0307 (6)	
H31	0.4494	−0.0092	0.8820	0.037*	
C32	0.1415 (3)	−0.0806 (2)	0.5837 (2)	0.0326 (6)	
C33	0.0246 (3)	−0.1084 (3)	0.5340 (3)	0.0512 (9)	
H33	−0.0127	−0.1060	0.5746	0.061*	
C34	−0.0376 (4)	−0.1393 (4)	0.4262 (3)	0.0562 (10)	
H34	−0.1167	−0.1572	0.3942	0.067*	
C35	0.0127 (4)	−0.1445 (3)	0.3657 (3)	0.0557 (10)	
H35	−0.0305	−0.1649	0.2921	0.067*	
C36	0.1252 (5)	−0.1203 (5)	0.4113 (3)	0.0714 (14)	
H36	0.1607	−0.1243	0.3692	0.086*	
C37	0.1893 (4)	−0.0894 (4)	0.5196 (3)	0.0577 (11)	
H37	0.2675	−0.0742	0.5499	0.069*	
O9	0.61719 (19)	0.23406 (19)	1.19859 (16)	0.0370 (5)	
N3	0.6813 (2)	0.24603 (19)	1.15242 (19)	0.0271 (5)	
C38	0.6372 (3)	0.2340 (3)	1.0509 (2)	0.0311 (6)	
H38	0.5584	0.2153	1.0116	0.037*	
C39	0.7061 (3)	0.2488 (3)	1.0046 (3)	0.0400 (8)	
H39	0.6746	0.2409	0.9336	0.048*	
C40	0.8210 (3)	0.2751 (3)	1.0604 (3)	0.0393 (7)	
C41	0.8631 (3)	0.2884 (3)	1.1643 (3)	0.0339 (7)	
H41	0.9422	0.3085	1.2054	0.041*	
C42	0.7933 (3)	0.2732 (2)	1.2091 (2)	0.0302 (6)	
H42	0.8238	0.2819	1.2803	0.036*	
C43	0.8729 (7)	0.2965 (6)	0.9206 (6)	0.0448 (7)*	0.50
H43A	0.8089	0.3056	0.8887	0.054*	0.50
C44	0.9464 (6)	0.3079 (6)	0.8798 (6)	0.0448 (7)*	0.50
H44A	0.9343	0.3273	0.8218	0.054*	0.50
C45	1.0363 (7)	0.2917 (6)	0.9213 (6)	0.0448 (7)*	0.50
H45A	1.0907	0.3056	0.8966	0.054*	0.50
C46	1.0471 (7)	0.2554 (7)	0.9985 (6)	0.0448 (7)*	0.50
H46A	1.1054	0.2379	1.0240	0.054*	0.50
C47	0.9733 (7)	0.2439 (6)	1.0402 (7)	0.0448 (7)*	0.50
H47A	0.9799	0.2161	1.0925	0.054*	0.50
C48	0.8902 (8)	0.2722 (6)	1.0071 (7)	0.0448 (7)*	0.50
C43'	0.9137 (9)	0.3762 (8)	0.9685 (8)	0.0666 (10)*	0.50
H43B	0.8620	0.4023	0.9552	0.080*	0.50
C44'	0.9954 (9)	0.4042 (8)	0.9330 (8)	0.0666 (10)*	0.50
H44B	0.9991	0.4485	0.8950	0.080*	0.50
C45'	1.0696 (9)	0.3658 (9)	0.9546 (8)	0.0666 (10)*	0.50
H45B	1.1208	0.3802	0.9263	0.080*	0.50
C46'	1.0736 (9)	0.3091 (9)	1.0137 (8)	0.0666 (10)*	0.50
H46B	1.1278	0.2857	1.0288	0.080*	0.50
C47'	0.9951 (10)	0.2852 (8)	1.0527 (9)	0.0666 (10)*	0.50
H47B	1.0006	0.2510	1.0999	0.080*	0.50
C48'	0.9089 (10)	0.3116 (8)	1.0220 (9)	0.0666 (10)*	0.50

### Atomic displacement parameters (Å^2^)

**Table:**

	*U*^11^	*U*^22^	*U*^33^	*U*^12^	*U*^13^	*U*^23^
Cu1	0.0264 (2)	0.0343 (3)	0.0234 (3)	0.0187 (2)	0.0131 (2)	0.0146 (2)
O1	0.0326 (11)	0.0436 (12)	0.0261 (11)	0.0249 (10)	0.0149 (9)	0.0162 (10)
C1	0.0253 (13)	0.0292 (14)	0.0295 (15)	0.0142 (12)	0.0133 (12)	0.0104 (12)
C2	0.0304 (15)	0.0407 (17)	0.0330 (17)	0.0197 (14)	0.0138 (13)	0.0107 (14)
F1	0.0718 (16)	0.0625 (14)	0.0680 (16)	0.0370 (13)	0.0562 (14)	0.0321 (13)
F2	0.0419 (11)	0.0450 (11)	0.0348 (11)	0.0236 (9)	0.0120 (9)	0.0031 (9)
F3	0.0518 (12)	0.0723 (15)	0.0465 (12)	0.0482 (12)	0.0207 (10)	0.0160 (11)
O2	0.0357 (11)	0.0385 (12)	0.0269 (11)	0.0231 (10)	0.0140 (9)	0.0144 (9)
C3	0.0256 (13)	0.0263 (14)	0.0255 (14)	0.0117 (12)	0.0077 (12)	0.0111 (12)
C4	0.0317 (15)	0.0383 (16)	0.0328 (16)	0.0235 (14)	0.0123 (13)	0.0151 (14)
C5	0.0373 (17)	0.0370 (17)	0.0305 (16)	0.0205 (14)	0.0126 (14)	0.0162 (14)
F4	0.112 (3)	0.301 (6)	0.114 (3)	0.139 (4)	0.085 (2)	0.166 (4)
F5	0.162 (3)	0.0783 (18)	0.0367 (13)	0.092 (2)	0.0167 (16)	0.0243 (13)
F6	0.090 (2)	0.0436 (13)	0.0457 (14)	0.0183 (14)	−0.0239 (14)	0.0079 (12)
O3	0.0348 (11)	0.0367 (12)	0.0356 (12)	0.0165 (10)	0.0206 (10)	0.0164 (10)
N1	0.0311 (13)	0.0357 (13)	0.0283 (13)	0.0148 (11)	0.0156 (11)	0.0167 (11)
C6	0.0318 (15)	0.0350 (16)	0.0268 (15)	0.0161 (13)	0.0116 (13)	0.0127 (13)
C7	0.0366 (16)	0.0369 (16)	0.0272 (15)	0.0189 (14)	0.0140 (13)	0.0132 (13)
C8	0.0399 (16)	0.0320 (15)	0.0280 (15)	0.0192 (13)	0.0176 (13)	0.0173 (13)
C9	0.0413 (19)	0.080 (3)	0.0310 (18)	0.027 (2)	0.0189 (16)	0.0317 (19)
C10	0.0324 (17)	0.083 (3)	0.0298 (18)	0.0220 (19)	0.0128 (15)	0.0302 (19)
C11	0.0450 (18)	0.0327 (15)	0.0347 (17)	0.0248 (14)	0.0238 (15)	0.0204 (14)
C12	0.046 (2)	0.054 (2)	0.044 (2)	0.0270 (18)	0.0278 (17)	0.0197 (18)
C13	0.056 (2)	0.073 (3)	0.069 (3)	0.034 (2)	0.043 (2)	0.028 (2)
C14	0.071 (3)	0.069 (3)	0.066 (3)	0.044 (2)	0.051 (3)	0.031 (2)
C15	0.080 (3)	0.051 (2)	0.047 (2)	0.044 (2)	0.044 (2)	0.0243 (19)
C16	0.058 (2)	0.0382 (17)	0.0382 (18)	0.0291 (17)	0.0282 (17)	0.0210 (15)
Cu2	0.03137 (19)	0.02930 (19)	0.02082 (18)	0.02031 (16)	0.01367 (15)	0.01207 (15)
O4	0.0349 (11)	0.0301 (10)	0.0224 (10)	0.0211 (9)	0.0147 (9)	0.0119 (8)
O5	0.0373 (11)	0.0387 (11)	0.0244 (10)	0.0258 (10)	0.0165 (9)	0.0144 (9)
C17	0.0382 (16)	0.0304 (15)	0.0285 (15)	0.0200 (13)	0.0141 (13)	0.0150 (13)
C18	0.0286 (14)	0.0288 (14)	0.0262 (14)	0.0166 (12)	0.0143 (12)	0.0134 (12)
C19	0.0420 (17)	0.0319 (15)	0.0320 (16)	0.0249 (14)	0.0212 (14)	0.0166 (13)
C20	0.0305 (14)	0.0324 (15)	0.0267 (15)	0.0187 (13)	0.0163 (12)	0.0104 (13)
C21	0.052 (2)	0.0425 (18)	0.0318 (17)	0.0295 (17)	0.0254 (16)	0.0138 (15)
F7	0.0542 (13)	0.0462 (12)	0.0382 (12)	0.0217 (11)	−0.0009 (10)	0.0199 (10)
F8	0.0859 (16)	0.0501 (12)	0.0448 (12)	0.0510 (12)	0.0322 (12)	0.0292 (10)
F9	0.0693 (15)	0.0824 (16)	0.0563 (14)	0.0482 (14)	0.0459 (13)	0.0500 (13)
F10	0.0552 (16)	0.0833 (19)	0.0497 (15)	0.0104 (14)	0.0199 (13)	−0.0245 (14)
F11	0.0966 (19)	0.0724 (16)	0.0618 (15)	0.0554 (15)	0.0632 (15)	0.0373 (13)
F12	0.149 (3)	0.0912 (19)	0.0620 (16)	0.100 (2)	0.0716 (19)	0.0442 (15)
O6	0.0355 (12)	0.0487 (13)	0.0371 (12)	0.0267 (11)	0.0193 (10)	0.0237 (11)
O7	0.0346 (11)	0.0402 (11)	0.0321 (11)	0.0263 (10)	0.0206 (9)	0.0216 (10)
C22	0.052 (2)	0.088 (3)	0.065 (3)	0.053 (2)	0.040 (2)	0.048 (3)
C23	0.0335 (16)	0.0385 (16)	0.0381 (17)	0.0237 (14)	0.0203 (14)	0.0170 (14)
C24	0.0424 (18)	0.054 (2)	0.052 (2)	0.0325 (17)	0.0327 (17)	0.0359 (18)
C25	0.0355 (15)	0.0317 (15)	0.0288 (15)	0.0203 (13)	0.0168 (13)	0.0159 (13)
C26	0.0460 (19)	0.050 (2)	0.050 (2)	0.0330 (17)	0.0304 (17)	0.0346 (18)
F13	0.245 (6)	0.244 (5)	0.264 (6)	0.229 (5)	0.227 (5)	0.213 (5)
F14	0.0376 (17)	0.271 (6)	0.132 (4)	0.060 (3)	0.018 (2)	−0.027 (4)
F15	0.125 (3)	0.208 (4)	0.123 (3)	0.139 (3)	0.107 (2)	0.121 (3)
F16	0.108 (2)	0.0641 (15)	0.0613 (15)	0.0694 (16)	0.0381 (15)	0.0358 (13)
F17	0.0657 (15)	0.0650 (15)	0.0434 (13)	0.0369 (13)	0.0113 (12)	0.0277 (12)
F18	0.0784 (18)	0.141 (3)	0.139 (3)	0.079 (2)	0.078 (2)	0.126 (2)
O8	0.0455 (13)	0.0359 (11)	0.0240 (11)	0.0285 (10)	0.0141 (10)	0.0116 (9)
N2	0.0331 (13)	0.0267 (12)	0.0228 (12)	0.0183 (11)	0.0114 (10)	0.0081 (10)
C27	0.0298 (14)	0.0328 (15)	0.0263 (15)	0.0173 (13)	0.0135 (12)	0.0065 (13)
C28	0.0267 (14)	0.0319 (15)	0.0274 (15)	0.0145 (12)	0.0098 (12)	0.0065 (13)
C29	0.0316 (15)	0.0262 (14)	0.0285 (15)	0.0137 (12)	0.0142 (13)	0.0102 (12)
C30	0.0358 (16)	0.0360 (16)	0.0319 (16)	0.0228 (14)	0.0200 (14)	0.0133 (13)
C31	0.0301 (14)	0.0313 (15)	0.0311 (16)	0.0188 (13)	0.0146 (13)	0.0100 (13)
C32	0.0352 (16)	0.0273 (14)	0.0274 (15)	0.0137 (13)	0.0132 (13)	0.0093 (13)
C33	0.043 (2)	0.067 (3)	0.0334 (19)	0.0256 (19)	0.0151 (16)	0.0177 (18)
C34	0.040 (2)	0.069 (3)	0.034 (2)	0.0219 (19)	0.0056 (16)	0.0168 (19)
C35	0.065 (3)	0.053 (2)	0.0250 (17)	0.023 (2)	0.0119 (18)	0.0151 (17)
C36	0.074 (3)	0.111 (4)	0.037 (2)	0.050 (3)	0.032 (2)	0.034 (3)
C37	0.049 (2)	0.086 (3)	0.038 (2)	0.036 (2)	0.0223 (18)	0.026 (2)
O9	0.0295 (11)	0.0481 (13)	0.0237 (11)	0.0139 (10)	0.0154 (9)	0.0096 (10)
N3	0.0291 (12)	0.0269 (12)	0.0227 (12)	0.0129 (10)	0.0143 (10)	0.0076 (10)
C38	0.0302 (15)	0.0383 (16)	0.0233 (14)	0.0194 (13)	0.0122 (12)	0.0104 (13)
C39	0.0434 (18)	0.059 (2)	0.0285 (16)	0.0326 (17)	0.0202 (15)	0.0212 (16)
C40	0.0394 (17)	0.056 (2)	0.0334 (17)	0.0295 (17)	0.0219 (15)	0.0228 (16)
C41	0.0301 (15)	0.0384 (16)	0.0311 (16)	0.0185 (13)	0.0133 (13)	0.0143 (14)
C42	0.0310 (14)	0.0298 (14)	0.0223 (14)	0.0142 (12)	0.0107 (12)	0.0075 (12)

### Geometric parameters (Å, °)

**Table:**

Cu1—O1^i^	1.966 (2)	C22—C23	1.539 (5)
Cu1—O1	1.966 (2)	C23—C24	1.417 (4)
Cu1—O2	2.331 (2)	C24—C25	1.374 (4)
Cu1—O2^i^	2.331 (2)	C24—H24	0.9500
Cu1—O3	1.968 (2)	C25—C26	1.535 (4)
Cu1—O3^i^	1.968 (2)	C26—F18	1.316 (4)
Cu2—O4	1.9532 (19)	C26—F16	1.328 (4)
Cu2—O5	1.990 (2)	C26—F17	1.332 (4)
Cu2—O6	2.365 (2)	O8—N2	1.338 (3)
Cu2—O7	1.975 (2)	N2—C31	1.343 (4)
Cu2—O8	1.961 (2)	N2—C27	1.355 (4)
Cu2—O9	2.230 (2)	C27—C28	1.369 (4)
O1—C1	1.263 (4)	C27—H27	0.9500
C1—C4^i^	1.376 (4)	C28—C29	1.403 (4)
C1—C2	1.527 (4)	C28—H28	0.9500
C2—F1	1.327 (4)	C29—C30	1.399 (4)
C2—F3	1.335 (4)	C29—C32	1.484 (4)
C2—F2	1.346 (4)	C30—C31	1.366 (4)
O2—C3	1.237 (4)	C30—H30	0.9500
C3—C4	1.402 (4)	C31—H31	0.9500
C3—C5	1.539 (4)	C32—C37	1.381 (5)
C4—C1^i^	1.376 (4)	C32—C33	1.398 (5)
C4—H4	0.9500	C33—C34	1.386 (5)
C5—F6	1.288 (4)	C33—H33	0.9500
C5—F4	1.296 (4)	C34—C35	1.355 (6)
C5—F5	1.298 (4)	C34—H34	0.9500
O3—N1	1.339 (3)	C35—C36	1.357 (7)
N1—C10	1.343 (4)	C35—H35	0.9500
N1—C6	1.344 (4)	C36—C37	1.396 (6)
C6—C7	1.376 (4)	C36—H36	0.9500
C6—H6	0.9500	C37—H37	0.9500
C7—C8	1.391 (4)	O9—N3	1.319 (3)
C7—H7	0.9500	N3—C42	1.347 (4)
C8—C9	1.385 (5)	N3—C38	1.356 (4)
C8—C11	1.487 (4)	C38—C39	1.377 (4)
C9—C10	1.367 (5)	C38—H38	0.9500
C9—H9	0.9500	C39—C40	1.386 (5)
C10—H10	0.9500	C39—H39	0.9500
C11—C12	1.384 (5)	C40—C41	1.383 (4)
C11—C16	1.398 (5)	C40—C48	1.493 (9)
C12—C13	1.396 (5)	C40—C48'	1.523 (12)
C12—H12	0.9500	C41—C42	1.370 (4)
C13—C14	1.378 (6)	C41—H41	0.9500
C13—H13	0.9500	C42—H42	0.9500
C14—C15	1.367 (7)	C43—C44	1.372 (10)
C14—H14	0.9500	C43—C48	1.380 (11)
C15—C16	1.389 (5)	C43—H43A	0.9500
C15—H15	0.9500	C44—C45	1.360 (10)
C16—H16	0.9500	C44—H44A	0.9500
O4—C18	1.262 (3)	C45—C46	1.358 (11)
O5—C20	1.249 (3)	C45—H45A	0.9500
C17—F9	1.323 (4)	C46—C47	1.387 (11)
C17—F8	1.327 (4)	C46—H46A	0.9500
C17—F7	1.336 (4)	C47—C48	1.384 (12)
C17—C18	1.532 (4)	C47—H47A	0.9500
C18—C19	1.374 (4)	C43'—C48'	1.363 (14)
C19—C20	1.396 (4)	C43'—C44'	1.408 (13)
C19—H19	0.9500	C43'—H43B	0.9500
C20—C21	1.535 (4)	C44'—C45'	1.373 (14)
C21—F12	1.306 (4)	C44'—H44B	0.9500
C21—F10	1.317 (5)	C45'—C46'	1.341 (14)
C21—F11	1.328 (4)	C45'—H45B	0.9500
O6—C23	1.224 (4)	C46'—C47'	1.407 (15)
O7—C25	1.259 (4)	C46'—H46B	0.9500
C22—F14	1.246 (6)	C47'—C48'	1.400 (16)
C22—F15	1.301 (5)	C47'—H47B	0.9500
C22—F13	1.307 (6)		
			
O1—Cu1—O2	93.37 (8)	C23—O6—Cu2	117.3 (2)
O1—Cu1—O2^i^	86.63 (8)	C25—O7—Cu2	127.78 (18)
O1—Cu1—O3	86.25 (9)	F14—C22—F15	108.1 (5)
O1—Cu1—O3^i^	93.75 (9)	F14—C22—F13	105.1 (5)
O3—Cu1—O2	93.08 (9)	F15—C22—F13	103.7 (4)
O3—Cu1—O2^i^	86.92 (9)	F14—C22—C23	113.8 (4)
O1^i^—Cu1—O1	180.000 (1)	F15—C22—C23	114.8 (3)
O1^i^—Cu1—O3	93.75 (9)	F13—C22—C23	110.5 (4)
O1^i^—Cu1—O3^i^	86.25 (9)	O6—C23—C24	128.7 (3)
O3—Cu1—O3^i^	180.000 (1)	O6—C23—C22	115.1 (3)
O1^i^—Cu1—O2	86.63 (8)	C24—C23—C22	116.2 (3)
O3^i^—Cu1—O2	86.92 (9)	C25—C24—C23	124.0 (3)
O1^i^—Cu1—O2^i^	93.37 (8)	C25—C24—H24	118.0
O3^i^—Cu1—O2^i^	93.08 (9)	C23—C24—H24	118.0
O2—Cu1—O2^i^	180.000 (1)	O7—C25—C24	130.6 (3)
O4—Cu2—O5	91.66 (8)	O7—C25—C26	111.7 (3)
O4—Cu2—O6	89.41 (8)	C24—C25—C26	117.6 (3)
O4—Cu2—O7	174.83 (9)	F18—C26—F16	108.3 (3)
O4—Cu2—O8	91.44 (8)	F18—C26—F17	106.8 (3)
O4—Cu2—O9	95.05 (9)	F16—C26—F17	104.4 (3)
O5—Cu2—O6	80.26 (9)	F18—C26—C25	114.7 (3)
O5—Cu2—O9	85.45 (9)	F16—C26—C25	110.7 (3)
O7—Cu2—O5	90.74 (9)	F17—C26—C25	111.3 (3)
O7—Cu2—O6	86.48 (8)	O8—N2—C31	118.6 (2)
O7—Cu2—O9	89.71 (9)	O8—N2—C27	120.6 (2)
O8—Cu2—O5	176.63 (9)	C31—N2—C27	120.7 (3)
O8—Cu2—O6	98.43 (9)	N2—C27—C28	120.1 (3)
O8—Cu2—O7	86.06 (9)	N2—C27—H27	120.0
O8—Cu2—O9	95.61 (9)	C28—C27—H27	120.0
O9—Cu2—O6	165.15 (8)	C27—C28—C29	121.4 (3)
N1—O3—Cu1	117.82 (18)	C27—C28—H28	119.3
N2—O8—Cu2	122.39 (16)	C29—C28—H28	119.3
N3—O9—Cu2	131.12 (17)	C30—C29—C28	115.7 (3)
C1—O1—Cu1	128.60 (19)	C30—C29—C32	122.1 (3)
O1—C1—C4^i^	131.0 (3)	C28—C29—C32	122.2 (3)
O1—C1—C2	111.9 (3)	C31—C30—C29	121.7 (3)
C4^i^—C1—C2	117.1 (3)	C31—C30—H30	119.2
F1—C2—F3	108.0 (3)	C29—C30—H30	119.2
F1—C2—F2	106.5 (3)	N2—C31—C30	120.4 (3)
F3—C2—F2	106.2 (3)	N2—C31—H31	119.8
F1—C2—C1	111.4 (3)	C30—C31—H31	119.8
F3—C2—C1	114.3 (3)	C37—C32—C33	116.6 (3)
F2—C2—C1	109.9 (3)	C37—C32—C29	121.9 (3)
C3—O2—Cu1	120.80 (19)	C33—C32—C29	121.4 (3)
O2—C3—C4	128.6 (3)	C34—C33—C32	121.0 (4)
O2—C3—C5	114.5 (3)	C34—C33—H33	119.5
C4—C3—C5	116.9 (3)	C32—C33—H33	119.5
C1^i^—C4—C3	123.8 (3)	C35—C34—C33	121.0 (4)
C1^i^—C4—H4	118.1	C35—C34—H34	119.5
C3—C4—H4	118.1	C33—C34—H34	119.5
F6—C5—F4	106.0 (4)	C34—C35—C36	119.4 (4)
F6—C5—F5	106.9 (3)	C34—C35—H35	120.3
F4—C5—F5	105.3 (4)	C36—C35—H35	120.3
F6—C5—C3	112.4 (3)	C35—C36—C37	120.5 (4)
F4—C5—C3	111.0 (3)	C35—C36—H36	119.7
F5—C5—C3	114.5 (3)	C37—C36—H36	119.7
O3—N1—C10	120.4 (3)	C32—C37—C36	121.4 (4)
O3—N1—C6	119.3 (2)	C32—C37—H37	119.3
C10—N1—C6	120.2 (3)	C36—C37—H37	119.3
N1—C6—C7	120.1 (3)	O9—N3—C42	118.6 (2)
N1—C6—H6	120.0	O9—N3—C38	121.1 (2)
C7—C6—H6	120.0	C42—N3—C38	120.3 (2)
C6—C7—C8	121.5 (3)	N3—C38—C39	120.3 (3)
C6—C7—H7	119.2	N3—C38—H38	119.9
C8—C7—H7	119.2	C39—C38—H38	119.9
C9—C8—C7	116.0 (3)	C38—C39—C40	120.6 (3)
C9—C8—C11	122.3 (3)	C38—C39—H39	119.7
C7—C8—C11	121.7 (3)	C40—C39—H39	119.7
C10—C9—C8	121.5 (3)	C41—C40—C39	117.2 (3)
C10—C9—H9	119.3	C41—C40—C48	120.6 (4)
C8—C9—H9	119.3	C39—C40—C48	121.6 (4)
N1—C10—C9	120.7 (3)	C41—C40—C48'	118.1 (5)
N1—C10—H10	119.7	C39—C40—C48'	123.6 (5)
C9—C10—H10	119.7	C42—C41—C40	121.4 (3)
C12—C11—C16	118.3 (3)	C42—C41—H41	119.3
C12—C11—C8	120.8 (3)	C40—C41—H41	119.3
C16—C11—C8	120.9 (3)	N3—C42—C41	120.2 (3)
C11—C12—C13	120.9 (4)	N3—C42—H42	119.9
C11—C12—H12	119.5	C41—C42—H42	119.9
C13—C12—H12	119.5	C44—C43—C48	121.1 (7)
C14—C13—C12	119.9 (4)	C44—C43—H43A	119.4
C14—C13—H13	120.0	C48—C43—H43A	119.4
C12—C13—H13	120.0	C45—C44—C43	121.0 (7)
C15—C14—C13	119.8 (4)	C45—C44—H44A	119.5
C15—C14—H14	120.1	C43—C44—H44A	119.5
C13—C14—H14	120.1	C46—C45—C44	119.0 (7)
C14—C15—C16	120.8 (4)	C46—C45—H45A	120.5
C14—C15—H15	119.6	C44—C45—H45A	120.5
C16—C15—H15	119.6	C45—C46—C47	120.1 (8)
C15—C16—C11	120.3 (4)	C45—C46—H46A	119.9
C15—C16—H16	119.9	C47—C46—H46A	119.9
C11—C16—H16	119.9	C48—C47—C46	121.2 (8)
C18—O4—Cu2	124.36 (18)	C48—C47—H47A	119.4
C20—O5—Cu2	123.79 (19)	C46—C47—H47A	119.4
F9—C17—F8	107.4 (3)	C43—C48—C47	116.6 (8)
F9—C17—F7	107.3 (3)	C43—C48—C40	121.0 (7)
F8—C17—F7	107.0 (3)	C47—C48—C40	122.3 (7)
F9—C17—C18	110.8 (2)	C48'—C43'—C44'	120.1 (10)
F8—C17—C18	113.6 (3)	C48'—C43'—H43B	120.0
F7—C17—C18	110.5 (2)	C44'—C43'—H43B	120.0
O4—C18—C19	129.1 (3)	C45'—C44'—C43'	118.3 (9)
O4—C18—C17	111.8 (2)	C45'—C44'—H44B	120.8
C19—C18—C17	119.1 (3)	C43'—C44'—H44B	120.8
C18—C19—C20	121.3 (3)	C46'—C45'—C44'	123.4 (10)
C18—C19—H19	119.4	C46'—C45'—H45B	118.3
C20—C19—H19	119.4	C44'—C45'—H45B	118.3
O5—C20—C19	128.5 (3)	C45'—C46'—C47'	118.0 (10)
O5—C20—C21	113.3 (3)	C45'—C46'—H46B	121.0
C19—C20—C21	118.1 (3)	C47'—C46'—H46B	121.0
F12—C21—F10	111.7 (3)	C48'—C47'—C46'	120.1 (10)
F12—C21—F11	106.3 (3)	C48'—C47'—H47B	119.9
F10—C21—F11	103.9 (3)	C46'—C47'—H47B	119.9
F12—C21—C20	113.6 (3)	C43'—C48'—C47'	119.3 (10)
F10—C21—C20	110.0 (3)	C43'—C48'—C40	123.3 (9)
F11—C21—C20	110.8 (3)	C47'—C48'—C40	117.1 (9)
			
O3—Cu1—O1—C1	−80.7 (3)	F14—C22—C23—O6	75.5 (6)
O3^i^—Cu1—O1—C1	99.3 (3)	F15—C22—C23—O6	−159.2 (4)
O2—Cu1—O1—C1	−173.6 (3)	F13—C22—C23—O6	−42.4 (5)
O2^i^—Cu1—O1—C1	6.4 (3)	F14—C22—C23—C24	−105.4 (6)
Cu1—O1—C1—C4^i^	−5.6 (5)	F15—C22—C23—C24	19.9 (6)
Cu1—O1—C1—C2	173.62 (19)	F13—C22—C23—C24	136.7 (4)
O1—C1—C2—F1	55.9 (4)	O6—C23—C24—C25	6.8 (6)
C4^i^—C1—C2—F1	−124.7 (3)	C22—C23—C24—C25	−172.2 (4)
O1—C1—C2—F3	178.7 (3)	Cu2—O7—C25—C24	2.2 (5)
C4^i^—C1—C2—F3	−1.9 (4)	Cu2—O7—C25—C26	−175.0 (2)
O1—C1—C2—F2	−62.0 (3)	C23—C24—C25—O7	8.2 (6)
C4^i^—C1—C2—F2	117.4 (3)	C23—C24—C25—C26	−174.7 (3)
O1^i^—Cu1—O2—C3	7.1 (2)	O7—C25—C26—F18	−177.2 (3)
O1—Cu1—O2—C3	−172.9 (2)	C24—C25—C26—F18	5.2 (5)
O3—Cu1—O2—C3	100.7 (2)	O7—C25—C26—F16	−54.3 (4)
O3^i^—Cu1—O2—C3	−79.3 (2)	C24—C25—C26—F16	128.0 (3)
Cu1—O2—C3—C4	−7.0 (4)	O7—C25—C26—F17	61.4 (4)
Cu1—O2—C3—C5	173.05 (18)	C24—C25—C26—F17	−116.3 (4)
O2—C3—C4—C1^i^	3.5 (5)	O4—Cu2—O8—N2	−16.7 (2)
C5—C3—C4—C1^i^	−176.5 (3)	O7—Cu2—O8—N2	158.7 (2)
O2—C3—C5—F6	77.2 (4)	O9—Cu2—O8—N2	−111.9 (2)
C4—C3—C5—F6	−102.8 (4)	O6—Cu2—O8—N2	72.9 (2)
O2—C3—C5—F4	−41.4 (4)	Cu2—O8—N2—C31	131.9 (2)
C4—C3—C5—F4	138.6 (4)	Cu2—O8—N2—C27	−52.0 (3)
O2—C3—C5—F5	−160.6 (3)	O8—N2—C27—C28	−175.8 (3)
C4—C3—C5—F5	19.5 (4)	C31—N2—C27—C28	0.2 (4)
O1^i^—Cu1—O3—N1	−9.2 (2)	N2—C27—C28—C29	2.0 (5)
O1—Cu1—O3—N1	170.8 (2)	C27—C28—C29—C30	−2.5 (4)
O2—Cu1—O3—N1	−95.98 (19)	C27—C28—C29—C32	177.3 (3)
O2^i^—Cu1—O3—N1	84.02 (19)	C28—C29—C30—C31	1.0 (4)
Cu1—O3—N1—C10	−73.5 (4)	C32—C29—C30—C31	−178.8 (3)
Cu1—O3—N1—C6	107.7 (3)	O8—N2—C31—C30	174.4 (3)
O3—N1—C6—C7	177.9 (3)	C27—N2—C31—C30	−1.7 (4)
C10—N1—C6—C7	−0.8 (5)	C29—C30—C31—N2	1.1 (5)
N1—C6—C7—C8	0.4 (5)	C30—C29—C32—C37	−9.3 (5)
C6—C7—C8—C9	0.1 (5)	C28—C29—C32—C37	170.9 (3)
C6—C7—C8—C11	−179.6 (3)	C30—C29—C32—C33	167.3 (3)
C7—C8—C9—C10	−0.1 (6)	C28—C29—C32—C33	−12.5 (5)
C11—C8—C9—C10	179.6 (4)	C37—C32—C33—C34	−2.2 (6)
O3—N1—C10—C9	−177.9 (4)	C29—C32—C33—C34	−179.0 (4)
C6—N1—C10—C9	0.8 (6)	C32—C33—C34—C35	0.6 (7)
C8—C9—C10—N1	−0.4 (7)	C33—C34—C35—C36	0.8 (7)
C9—C8—C11—C12	−155.9 (4)	C34—C35—C36—C37	−0.5 (8)
C7—C8—C11—C12	23.8 (5)	C33—C32—C37—C36	2.5 (6)
C9—C8—C11—C16	23.4 (5)	C29—C32—C37—C36	179.3 (4)
C7—C8—C11—C16	−157.0 (3)	C35—C36—C37—C32	−1.2 (8)
C16—C11—C12—C13	−0.1 (5)	O4—Cu2—O9—N3	−55.3 (3)
C8—C11—C12—C13	179.1 (3)	O8—Cu2—O9—N3	36.6 (3)
C11—C12—C13—C14	−0.8 (7)	O7—Cu2—O9—N3	122.6 (2)
C12—C13—C14—C15	1.4 (7)	O5—Cu2—O9—N3	−146.6 (3)
C13—C14—C15—C16	−1.1 (6)	O6—Cu2—O9—N3	−162.3 (3)
C14—C15—C16—C11	0.2 (5)	Cu2—O9—N3—C42	−158.7 (2)
C12—C11—C16—C15	0.4 (5)	Cu2—O9—N3—C38	22.9 (4)
C8—C11—C16—C15	−178.9 (3)	O9—N3—C38—C39	178.8 (3)
O8—Cu2—O4—C18	168.4 (2)	C42—N3—C38—C39	0.4 (4)
O5—Cu2—O4—C18	−10.3 (2)	N3—C38—C39—C40	0.6 (5)
O9—Cu2—O4—C18	−95.8 (2)	C38—C39—C40—C41	−1.6 (5)
O6—Cu2—O4—C18	70.0 (2)	C38—C39—C40—C48	169.0 (4)
O4—Cu2—O5—C20	11.7 (2)	C38—C39—C40—C48'	−169.2 (6)
O7—Cu2—O5—C20	−163.7 (2)	C39—C40—C41—C42	1.8 (5)
O9—Cu2—O5—C20	106.7 (2)	C48—C40—C41—C42	−169.0 (4)
O6—Cu2—O5—C20	−77.4 (2)	C48'—C40—C41—C42	170.0 (5)
Cu2—O4—C18—C19	4.9 (4)	O9—N3—C42—C41	−178.7 (3)
Cu2—O4—C18—C17	−174.18 (18)	C38—N3—C42—C41	−0.3 (4)
F9—C17—C18—O4	−60.0 (3)	C40—C41—C42—N3	−0.8 (5)
F8—C17—C18—O4	179.0 (3)	C48—C43—C44—C45	2.3 (12)
F7—C17—C18—O4	58.8 (3)	C43—C44—C45—C46	5.2 (12)
F9—C17—C18—C19	120.9 (3)	C44—C45—C46—C47	−5.3 (12)
F8—C17—C18—C19	−0.1 (4)	C45—C46—C47—C48	−2.1 (12)
F7—C17—C18—C19	−120.4 (3)	C44—C43—C48—C47	−9.3 (12)
O4—C18—C19—C20	3.6 (5)	C44—C43—C48—C40	171.4 (7)
C17—C18—C19—C20	−177.4 (3)	C46—C47—C48—C43	9.3 (12)
Cu2—O5—C20—C19	−8.0 (5)	C46—C47—C48—C40	−171.5 (7)
Cu2—O5—C20—C21	172.2 (2)	C41—C40—C48—C43	−157.6 (6)
C18—C19—C20—O5	−1.7 (5)	C39—C40—C48—C43	32.1 (9)
C18—C19—C20—C21	178.1 (3)	C48'—C40—C48—C43	−70 (2)
O5—C20—C21—F12	−163.7 (3)	C41—C40—C48—C47	23.3 (9)
C19—C20—C21—F12	16.5 (5)	C39—C40—C48—C47	−147.1 (7)
O5—C20—C21—F10	70.2 (4)	C48'—C40—C48—C47	111 (2)
C19—C20—C21—F10	−109.7 (3)	C48'—C43'—C44'—C45'	−0.7 (15)
O5—C20—C21—F11	−44.1 (4)	C43'—C44'—C45'—C46'	−4.5 (16)
C19—C20—C21—F11	136.0 (3)	C44'—C45'—C46'—C47'	1.9 (16)
O4—Cu2—O6—C23	−160.1 (2)	C45'—C46'—C47'—C48'	5.7 (16)
O8—Cu2—O6—C23	108.5 (2)	C44'—C43'—C48'—C47'	8.0 (16)
O7—Cu2—O6—C23	23.0 (2)	C44'—C43'—C48'—C40	−178.3 (9)
O5—Cu2—O6—C23	−68.3 (2)	C46'—C47'—C48'—C43'	−10.6 (16)
O9—Cu2—O6—C23	−52.4 (4)	C46'—C47'—C48'—C40	175.2 (9)
O8—Cu2—O7—C25	−112.3 (3)	C41—C40—C48'—C43'	−131.0 (9)
O5—Cu2—O7—C25	66.6 (3)	C39—C40—C48'—C43'	36.5 (12)
O9—Cu2—O7—C25	152.0 (3)	C41—C40—C48'—C47'	42.9 (11)
O6—Cu2—O7—C25	−13.6 (3)	C39—C40—C48'—C47'	−149.7 (8)
Cu2—O6—C23—C24	−23.4 (5)	C48—C40—C48'—C47'	−59.9 (19)
Cu2—O6—C23—C22	155.6 (3)		

Symmetry codes: (i) −*x*+2, −*y*+1, −*z*+1.

### Hydrogen-bond geometry (Å, °)

**Table:**

*D*—H···*A*	*D*—H	H···*A*	*D*···*A*	*D*—H···*A*
C41—H41···F2^ii^	0.95	2.45	3.219 (5)	138
C45'—H45B···F4^i^	0.95	2.31	3.159 (2)	148
C42—H42···O3^ii^	0.95	2.47	3.376 (3)	160
C6—H6···O9^iii^	0.95	2.27	3.215 (3)	171
C31—H31···O8^iv^	0.95	2.41	3.333 (5)	163
C27—H27···O6	0.95	2.55	3.389 (4)	147
C38—H38···O4	0.95	2.55	3.334 (6)	140
C10—H10···O2^i^	0.95	2.51	3.249 (6)	134

Symmetry codes: (ii) *x*, *y*, *z*+1; (i) −*x*+2, −*y*+1, −*z*+1; (iii) *x*, *y*, *z*−1; (iv) −*x*+1, −*y*, −*z*+2.
